# Mini-implants and miniplates generate sub-absolute and absolute anchorage

**DOI:** 10.1590/2176-9451.19.3.020-023.oin

**Published:** 2014

**Authors:** Alberto Consolaro

**Affiliations:** 1 Full professor, School of Dentistry - University of São Paulo/Bauru and professor at the postgraduate program at the School of Dentistry -University of São Paulo/ Ribeirão Preto.

**Keywords:** Miniplates, Mini-implants, Osteocytes, Mechanotransduction, Periosteum, Orthopedics

## Abstract

The functional demand imposed on bone promotes changes in the spatial properties of osteocytes as well as in their extensions uniformly distributed throughout the mineralized surface. Once spatial deformation is established, osteocytes create the need for structural adaptations that result in bone formation and resorption that happen to meet the functional demands. The endosteum and the periosteum are the effectors responsible for stimulating adaptive osteocytes in the inner and outer surfaces.Changes in shape, volume and position of the jaws as a result of skeletal correction of the maxilla and mandible require anchorage to allow bone remodeling to redefine morphology, esthetics and function as a result of spatial deformation conducted by orthodontic appliances. Examining the degree of changes in shape, volume and structural relationship of areas where mini-implants and miniplates are placed allows us to classify mini-implants as devices of subabsolute anchorage and miniplates as devices of absolute anchorage.

## Abstract (pt)

Uma demanda funcional sobre o osso promove alterações na forma espacial da rede de osteócitos e seus prolongamentos, distribuídos uniformemente na estrutura mineralizada. A partir da deformação espacial captada, os osteócitos comandam a necessidade de adaptações estruturais, formando osso em novas áreas e reabsorvendo em outras, para que sejam atendidas as demandas funcionais. O endósteo e o periósteo são os verdadeiros efetores desses estímulos osteocíticos adaptativos, nas superfícies internas e externas. As alterações de forma, volume e posição dos ossos maxilares, nas correções esqueléticas da maxila e mandíbula, requerem uma ancoragem para que a remodelação óssea redefina a morfologia, a estética e as funções, a partir de deformações espaciais dirigidas por aparelhos. Verificar o grau de alterações na forma, volume e relações estruturais das áreas onde se fixaram os mini-implantes e as miniplacas poderá levar à classificação dos mini-implantes como dispositivos de ancoragem subabsoluta e as miniplacas, como de ancoragem absoluta.

The protein cytoskeleton of cells is responsible for maintaining normal tridimensional cell shape, as well as cell movement and migration. Cytoskeletal proteins are classified according to their molecular weight and spatial structure as: microtubules, microfilaments and intermediate filaments.

In all body systems, the balance provided by the intrinsic annulation of all forces results in a force equals to zero known as tensegrity. All cells tend to be similar in shape as a result of balance established between inner and outer forces that, in turn, result from a mutual annulation between them. This state of balance or stability is also known as cellular tensegrity.

Whenever tensegrity is lost by compression of the cytoskeleton, the latter tends to go back to its natural state similarly to other natural systems, but by stimulating a set of events so as to meet that purpose. Chemical mediators are released to induce cell and tissue phenomena, which is part of the process established by the cells with a view to restoring tensegrity. Tensegrity is responsible for determining stability of shape and standard morphology of an object or system, particularly cells.

Breaking tensegrity affects the permeability of cell membrane and results in activation of intracellular metabolic pathways with release of substances that act as mediators capable of inducing cellular, tissue and/or vascular phenomena. These substances are the cytokines, growth factors and products of arachidonic acid. This mechanism transforms a physical event, such as force, into biological and biochemical events. This transformations is also known as mechanotransduction.

## Osteocytes are mechanotransductors!

**Figure 1:**
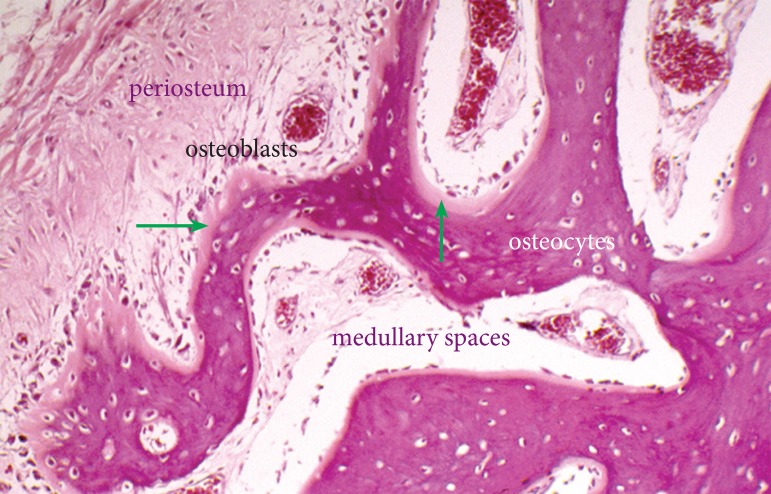
Osteocytes are the most numerous cells of the skeleton. Acting as mechanotransductors, they are able to pick up signs of minor structural deformations. Polyhedron-shaped osteoblasts are arranged in palisade in the surface of trabecular and cortical bone. The arrows indicate the osteoid which represents the last recently-deposited, non-mineralized bone layer. (HE, 40X).

**Figure 2:**
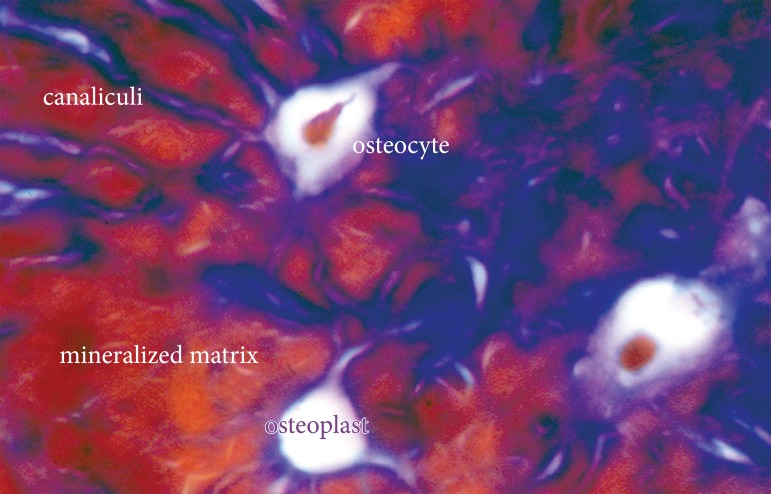
In the mineralized bone matrix, osteocytes have many cytoplasmic extensions that interconnect with other 40 to 50 cells and, three-dimensionally, capture minor structural deformations. They fill the lacunae known as osteoplasts and their extensions are distributed in canaliculi filled with a tissue fluid that carries mediators (Mallory, 100X).

Osteocytes have between 40 and 50 extensions and, for this reason, have a dendritic shape.^11^ They comprise 90 to 95% of adult bone cells^15^ and are included in mineralized bone matrix inside the lacunae also known as osteoplasts (Figs 1, 2). Osteocyte extensions allow osteocytes to communicate with each other and with bone surface cells. The extensions are distributed in 100 to 300-nm thick canaliculi^3,4,5^ that, three-dimensionally, form a network that resembles the neural network of the central nervous system. The canaliculi are filled with a tissue fluid that carries nutrients as well as mediators and connects the osteocytes, not only with the cells of the cortical and trabecular surfaces, but also with bone marrow cells.^10^

This network captures potential bone deformations resulting from compression and traction deflection. The osteocyte network acts as an excellent mechanotransductor. Additionally, it also plays a major role in bone metabolism by releasing mediators that reach the bone surface.^16,17^

## Periosteum and endosteum act!

The surface of bone trabeculae has a thin layer of connective tissue that consists of osteoblasts and undifferentiated cells and functions as bone modeling units and its clasts: the endosteum. It has a superior osteogenic and resorptive capacity that meets the increasing demand for bone remodeling.

Similarly, the outer surface of cortical bone is lined by the periosteum, a thicker membrane of fibrous connective tissue that covers outer bone surface. The outer periosteum layer is fibrous; whereas its inner layer, which touches the cortex, is highly cellularized and vascularized with young as well as pre and undifferentiated cells. These characteristics provide the periosteum with a high osteogenic reactional capacity.

The osteocytes network form a very sensitive 3D system that uptakes bone deformities. Any change in bone form during skeleton function can be captured by this sensitive network/web of osteocytes and extensions or mechanotransduction detection system. Exercise can increase bone structure by initially mechanical stimuli on this strain capturing network.

In other words: alterations in bone volume and shape are captured by the tridimensional osteocyte network that releases mediators and stimuli that allow osteoblasts and clasts to form or resorb bone according to the need for adaptation established by functional demand. Functional demand refers to alterations in shape and volume induced by tension and compression exerted by the action of orthodontic and orthopedic appliances, similarly to what occurs with natural body movements.

## Osteocytes as mechanotransductors: more details

Osteocytes individually pick up signals by mechanical deformation of their cytoskeleton. At the same time, the network in which each osteocyte participates, distributed throughout the bone structure, picks up deformations, overloads, deflections and limitations of nutrients. Deformation of the cytoskeleton as well as restriction of oxygen and nutrients stress the osteocytes. As a result, osteocytes release mediators to communicate with each other, as well as with osteoblasts and clasts on the bone surface, inducing them to reactive or adaptive phenomena.

During orthodontic movement, osteocytes undergo mechanical stress, which increases the production of mediators that circulate through the fluid in the canaliculi. Mediators reach the respective periodontal and bone surfaces and stimulate or inhibit bone formation and/or bone resorption in the "distant" cortical bone surface. In the bone marrow inside the bone, these mediators can influence higher or lower production of clastic cells and osteoclastogenesis.

Therefore, osteocytes strongly influence the function of bone to adapt its shape according to the determination of functional demands, thereby changing mechanical stimuli into biochemical events, a phenomenon also known as mechanotransduction.^2,7,13^Osteocytes also play a major role in regulating mineral metabolism,^9^ in addition to inducing changes in the properties of bone matrix around it.^12^ However, these functions were already well known.

The skeleton is able to continuously adapt to mechanical loads by adding new bone so as to increase the ability to resist or remove bone in response to a lighter load or lack of use.^6,8^ Osteocytes have high interconnectivity and are considered as bone mechanotransductors. Osteocytes increase glucose-6-dehydrogenase phosphatase after a few minutes of load.^19^ This enzyme is a marker of increased metabolism which occurs in cells associated with bone surface. Seconds after load is applied on the osteocytes; nitric oxide, prostaglandins and other molecules, such as ATP, increase.^1^

Therefore, when facing induced loads, osteocytes have the ability to release mediators that stimulate the precursors of clasts or osteoclastogenesis to differentiate into new clasts, increasing the rate of resorption. Among these mediators, the M-CSF, or stimulating factor of colonies for macrophages, and the RANKL are the most significant ones.^14^ It can be argued that osteocytes can command the activities of the clasts on bone surfaces according to functional demand. The set of osteocytes or the lacunocanalicular osteocyte system can be considered as a real endocrine body.^4^

## Correction of maxillary and skeletal alterations; miniplates and mini-implants

Tooth movement associated with changes in bone position, volume and shape continuously changes, for weeks or months, the tridimensional shape of maxillary bone. Absolute anchorage is required for these deformations to be efficient.

The use of miniplates^18^ provides enough anchorage to change the osteocyte network, causing it to release mediators that induce osteoblasts and clasts to directly reshape and restore bone volume and structure. Bone shape responds to functional demand and is able to correct major skeletal alterations, which not long ago was only possible through surgery.

The use of mini-implants with a view to causing major changes in shape, volume and dentoskeletal relationship is limited. Mini-implants are usually placed in the alveolar process of the maxilla and/or mandible or near them. Mini-implant anchorage may result in deflection in its placement sites, which decreases the "absolute" anchorage mini-implants provide within a limited system of force.

If mini-implants require greater force for correction of skeletal alterations, we can say that they offer subabsolute anchorage. As for miniplates, they offer real absolute anchorage as a result of being fixed in upper areas such as thicker cortical bone and denser trabeculae. Proper anchorage and thicker bone structure hardly allow deflection and deformation of the osteocyte network, thus providing absolute anchorage.

## Final considerations

This insight recommends some studies that can be used to examine the degree of changes in shape, volume and structure in the areas where mini-implants and miniplates are placed for anchorage necessary for tooth movement and associated skeletal correction. Such studies allow us to classify mini-implants as devices of subabsolute anchorage and miniplates as devices of absolute anchorage.
